# Computational SNP Analysis and Molecular Simulation Revealed the Most Deleterious Missense Variants in the NBD1 Domain of Human ABCA1 Transporter

**DOI:** 10.3390/ijms21207606

**Published:** 2020-10-14

**Authors:** Raju Dash, Md. Chayan Ali, Md. Liton Rana, Yeasmin Akter Munni, Largess Barua, Israt Jahan, Mst. Fatema Haque, Md. Abdul Hannan, Il Soo Moon

**Affiliations:** 1 Department of Anatomy, Dongguk University College of Medicine, Gyeongju 38066, Korea; rajudash.bgctub@gmail.com (R.D.); yeasminakteracce@gmail.com (Y.A.M.); hannanbmb@bau.edu.bd (M.A.H.); 2 Department of Biotechnology and Genetic Engineering, Faculty of Biological Sciences, Islamic University, Kushtia 7003, Bangladesh; chayanali7@gmail.com (M.C.A.); litonrana2050@gmail.com (M.L.R.); fatemahaque19@gmail.com (M.F.H.); 3 Department of Pharmacy, BGC Trust University Bangladesh, Chittagong 4381, Bangladesh; largessvishal@gmail.com; 4 Department of Pharmacy, Faculty of Life and Earth Sciences, Jagannath University, Dhaka 1100, Bangladesh; jahaanisrat6@gmail.com; 5 Department of Biochemistry and Molecular Biology, Bangladesh Agricultural University, Mymensingh 2202, Bangladesh

**Keywords:** ABCA1, NBD1, nsSNPs, polymorphism, molecular dynamics simulations, in silico

## Abstract

The ATP-binding cassette transporter A1 (ABCA1) is a membrane-bound exporter protein involved in regulating serum HDL level by exporting cholesterol and phospholipids to load up in lipid-poor ApoA-I and ApoE, which allows the formation of nascent HDL. Mutations in the *ABCA1* gene, when presents in both alleles, disrupt the canonical function of ABCA1, which associates with many disorders related to lipid transport. Although many studies have reported the phenotypic effects of a large number of ABCA1 variants, the pathological effect of non-synonymous polymorphisms (nsSNPs) in ABCA1 remains elusive. Therefore, aiming at exploring the structural and functional consequences of nsSNPs in *ABCA1*, in this study, we employed an integrated computational approach consisting of nine well-known in silico tools to identify damaging SNPs and molecular dynamics (MD) simulation to get insights into the magnitudes of the damaging effects. In silico tools revealed four nsSNPs as being most deleterious, where the two SNPs (G1050V and S1067C) are identified as the highly conserved and functional disrupting mutations located in the NBD1 domain. MD simulation suggested that both SNPs, G1050V and S1067C, changed the overall structural flexibility and dynamics of NBD1, and induced substantial alteration in the structural organization of ATP binding site. Taken together, these findings direct future studies to get more insights into the role of these variants in the loss of the ABCA1 function.

## 1. Introduction

The ATP-binding cassette transporter A1 (ABCA1) is a 220 kDa exporter protein from the ATP-binding cassette (ABC) family [1,2], which is expressed in diverse tissues, including peripheral blood leukocytes, intestinal, liver, adrenal glands, macrophages, and lungs [3,4,5]. The expressing gene, *ABCA1*, spans over 150 kb, located on the chromosome 9q22-q31, and contains about 50 exons and 49 introns [6]. The gene expresses upon the activation of retinoid X receptor and nuclear X receptor in response to oxysterols and various synthetic ligands [7], which is highly dominated by intracellular cholesterol changes, lipid loading via nuclear receptor and pro-oxidant substances [8]. Extensive evidence suggests that ABCA1 plays a critical role in reverse cholesterol transport by HDL biogenesis, where HDL facilitates cholesterol transportation to the liver from the peripheral tissues and thus inhibits foam cell formation and atherosclerosis [9,10]. ABCA1 exerts a rate-controlling step in HDL biogenesis through exporting cholesterol and phospholipids to load up in lipid-poor ApoA-I and ApoE and thus is enabled to form nascent HDL [11,12,13,14]. The reduced ABCA1 activity can cause cholesteryl esters (CE) accumulation, block the transformation of lipid-poor ApoA-I particles into pre-β HDL, and enhance rapid catabolism of poorly lapidated ApoA-I [15]. In contrast, lack of ABCA1 in the brain causes a substantial fall in neuronal ApoE levels [16].

Until the present, about 97 mutations have been found in the *ABCA1* gene, and a large number of them are linked with Tangier disease (TD), which is described as the accretion of cholesterol in reticuloendothelial cells that increases the risk of coronary heart disease [17,18,19]. In addition to Tangier disease [20,21], variations and alterations in the *ABCA1* gene enhance the risk of allelic disorders, such as atherosclerosis [22,23], ischemic stroke [23,24], coronary heart disease [25], type II diabetes [26,27,28], myocardial infarction (MI) [6], familial hypercholesterolaemia (FH) [29], cancer [30,31,32], Alzheimer’s disease [33], and macular degeneration [34]. Mutations in the *ABCA1* gene also increase the incidence of systemic and plaque inflammation [35]. 

Genetic variations in the *ABCA1* gene result in defective proteins, which cause the loss of substrate binding and various impairments such as impaired ABCA1 trafficking and transport activity, disrupt Apo-AI binding, impaired ATP binding/hydrolysis, and thus disrupts the normal cellular functions. Despite ample evidence supporting the role of ABCA1 variants in common age-associated disorders, there is a lack of information on the non-synonymous polymorphisms (nsSNPs) effect on ABCA1 structure and function. It has been ascertained that nsSNP influences approximately 60% of Mendelians diseases by altering phenotypic expression. Studies showed that genetic variation in ABC transporter genes responsible for various human diseases with Mendelian or complex inheritance [36,37], where it is found that SNPs in the *ABCA1* gene with rare allele frequency >1% affect plasma HDL levels in the general population [38]. Therefore, it is crucial to identify the comparison between disease-associated variants with neutral variants, which would clarify the genotype–phenotype relationship of diseases, assist in considering the most appropriate candidates for population association studies, and aid in developing diagnostic kits and drugs [39,40]. However, applying in vitro experimental approach would be more critical to discriminate between pathogenic and neutral variants [41] because of the high polymorphic nature of *ABCA1* gene [42]. In such a case, employing bioinformatics-based screening together with molecular dynamics (MD) simulation would provide critical insight into the deleterious effects of nsSNPs while prioritizing candidate nsSNP for further experimental characterization.

Taking this fact into consideration, the structural and functional implications of nsSNPs of ABCA1 have been evaluated in this study using various computational approaches, including MD simulation. Therefore, bioinformatic prediction tools have become extremely critical for the initial analysis of their molecular functions and prioritizing further experimental characterization, including deciphering the effects of ABCA1 SNPs [43]. Based on these approaches, the present study identified two potential deleterious nsSNPs in *ABCA1* (G1050V and S1067C), which are located in the NBD1 domain. Both of these nsSNPs appeared to disrupt NBD1 conformational flexibility and altered the structural organization of the catalytic site, which could have implications in ATP hydrolysis. 

## 2. Results

### 2.1. Dataset Selection and Screening 

**Figure 1:**
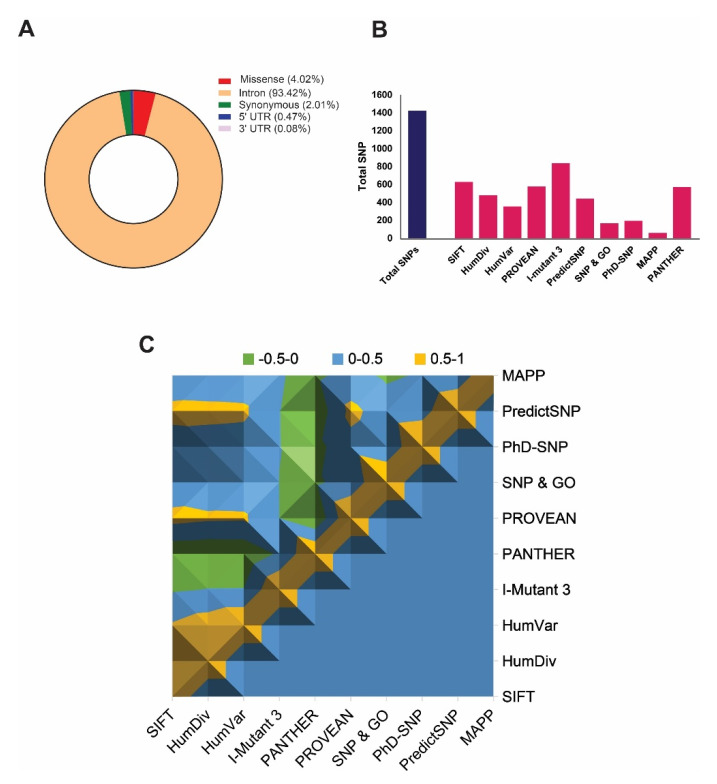
Graphical representation highlighting the distribution of total single-nucleotide polymorphisms (SNPs) and deleterious non-synonymous SNPs (nsSNPs) in the *ABCA1* gene. (**A**) Distribution of SNPs in the *ABCA1* gene, including missense, synonymous, intron, 5′UTR, and 3′UTR regions. (**B**) The plot describes the total number of predicted deleterious nsSNPs in *ABCA1* gene by using various state-of-the-art predictors. (**C**) Surface chart representing correlations among the damaging SNP prediction by all tools used for *ABCA1* gene.

The NCBI dbSNP database [44] contains a total of 35,328 single-nucleotide polymorphisms (SNPs) related to the ABCA1 gene, and among them, *Homo sapiens* is annotated by 37.5329% of total SNPs, as of January 2020. These includes 4.02% (1421 SNPs) missense variants, 93.42% (33002 SNPs) in intron, 0.473% (167SNPs) in 5’ UTR-untranslated region, 0.08% (28 SNPs) in 3’ UTR region, and 2.01% (710 SNPs) in coding synonymous region (Figure 1A). We considered only missense SNPs in this study for detailed analysis. 

### 2.2. Identification of Most Deleterious nsSNPs

A total number of 1421 SNPs has been screened out to identify highly deleterious SNPs by accrediting the nine most widely accepted deleterious SNP predictors, including SIFT (≤0.05), polyphen-2 HumDiv (˃0.9), Polyphen-2 HumVar (˃0.9), PROVEAN (≤−2.5), I-Mutant 3.0 (<−0.5), PhD-SNP (>0.5), SNP & GO (>0.5), PANTHER (≥0.5), PredictSNP, and MAPP, where parenthesis show the cut-offs used to predict deleteriousness (Supplementary File 1). The results show that the prediction from all the tools was significantly correlated (*p* < 0.0001) with each other (Figure 1C) when the predictions from any of the two tools were being compared. However, the total number of identified deleterious SNPs was varied by each tool. Figure 1B shows the total number of damaging SNPs predicted from each tool, where I-Mutant 3.0 showed the highest number of deleterious SNPs. Cumulatively, results from all tools revealed S741C as the most damaging nsSNP (*p* = 1.0 × 10^−90^), whereas three other nsSNPs, including Y793C, G1050V, and S1067C, were identified as the highly damaging SNPs, which were predicted as deleterious in all tools except SNP & GO and MAPP (Table 1). Hence, we considered these 4 nsSNPs for further analysis. 

### 2.3. Evolutionary Conservancy 

**Figure 2:**
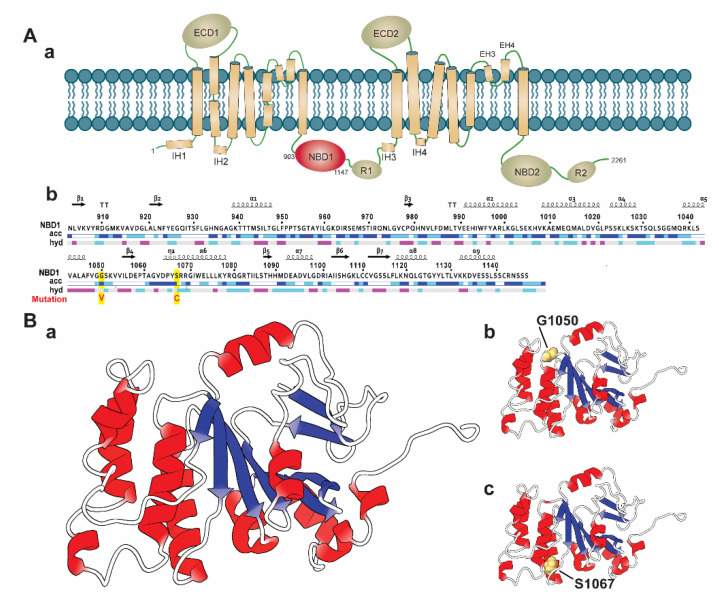
Molecular architecture of ABCA1 and sequence with the three-dimensional structure of the NBD1 domain labeled with wild and identified variants. (**A**) Schematic diagram representing the domain arrangement of ABCA1 protein highlighting the NBD1 domain (in red color) (**a**). The full sequence of NBD1 domain (903 to 1146), labeled with variant positions (in red, highlighted yellow) (**b**). (**B**) Three-dimensional representation of the ABCA1 NBD1 domain in a cartoon model, which is designated as wild-type (**a**), where the variant positions examined in this study are shown (**b**,**c**), representing G1050 and S1067, respectively. The model of the ABCA1 NBD1 domain was rendered using the PDB id: 5XJY [50].

It is evidenced that SNPs located in the conserved region are highly deleterious than those located in the non-conserved region in a protein [45]. Therefore, we considered the Consurf tool to analyze the degree of the conservancy of the selected highly deleterious four nsSNPs, including S741C, Y793C, G1050V, and S1067C. The Consurf tool identified that the residue position G1050 and S1067 of G1050V and S1067C, are highly conserved than the other nsSNPs (S741C and Y793C), having a conservation score of 8–9. Remarkably, G1050 and S1067 are identified with functional importance, in which G1050 is exposed to a solvent, whereas S1067 is buried in protein structure (Supplementary File 2). Structurally, ABCA1 is composed of multiple domains, including two extracellular domains (ECDs), two transmembrane domains (TMDs), and two nucleotide-binding domains (NBDs) (Figure 2(Aa)) [19,46]. Among these domains, ECDs are accountable for accommodating Apo-I [47], which have a regulatory function and maintain protein-protein interactions [48]. On the other hand, TMD domains create hydrophobic channels to provide space for substrate translocation. They make substrate-binding proteins (SBPs) docking sites in ABCA1 uptake systems and transfer a signal to the NBDs to bind and hydrolyze ATP [48]. The NBD domains are the most conserved domains among the ABC subfamily and are connected with small regulatory areas (R) [19,49]. These domains are called engines of ABCA1 receptors proteins, as they supply power to it and translocate the substrate through the binding and hydrolysis of ATP within the areas [48]. Interestingly, both nsSNPs (G1050V and S1067C) are located in the NBD1 domain (residue 903 to 1147, as shown in Figure 2(Ab)), suggesting that these nsSNPs may involve in the functional disruption of the domain by disrupting ATP binding process and ATP hydrolysis in the coupling of TMDs conformational change. Hence, we systematically analyzed the deleterious effects of G1050V and S1067C (Figure 2(Bb,C)) in the ABCA1 NBD1 domain by using MD simulation. 

### 2.4. Molecular Dynamics (MD) Simulation 

To assess the effect of nsSNPs in the dynamic and structural stability of the NBD1 domain of ABCA1, we performed MD simulation for 300 ns for both wild-type and variants (G1050V and S1067C). The simulated trajectories were initially considered for RMSD analysis, which denotes the conformation stability of the protein during the simulation. RMSD analysis represented that all proteins, including the wild and variants, reached equilibration after 120 ns of simulation and maintained stability afterward (Figure S1, Supplementary File 3). Therefore, the last 180 ns trajectories for all systems counting wild, G1050V, and S1067C were considered for detailed analysis. 

#### 2.4.1. Effects of Variants on Conformational Dynamics

**Figure 3:**
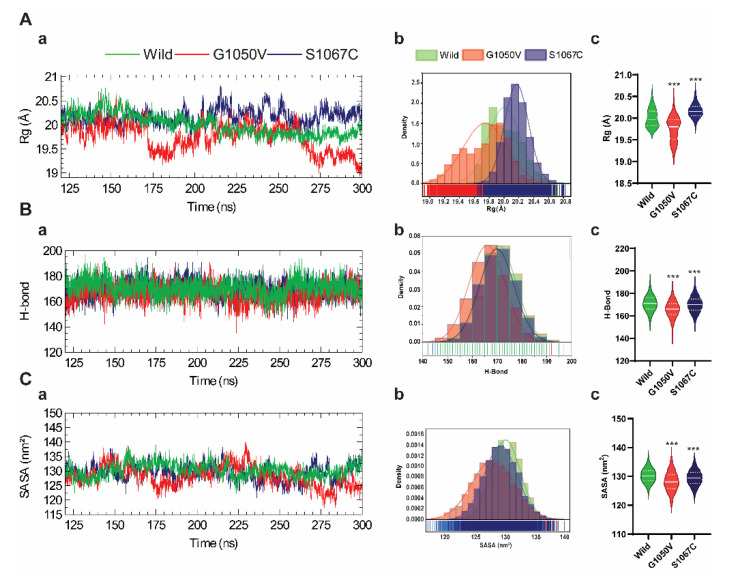
Effect of variants on the protein conformational stability of the NBD1 domain. (**A**) Calculation of radius of gyration (Rg), which is represented as a time-dependent change during the simulation (**a**), probability density (**b**), and the violin plot is highlighting the Rg distribution of each system (**c**). (**B**) Calculation of total intra-residue hydrogen bond represented as a time-dependent change (**a**), probability density (**b**), and the violin plot is highlighting the distribution of total hydrogen bond for the individual system (**c**). (**C**) Calculation of total solvent accessible surface area (SASA), highlighted as a time-dependent change (**a**), probability density (**b**), and the violin plot highlighting the distribution of SASA for each system (**c**). In every case, color in all plots, that is, green, red, dark blue, describes wild, G1050V, and S1067C, respectively. Violin plot highlights the first, median (second), and third quartiles, where the frequency of occurrence corresponds to the width of the box. The annotations used in A-c, B-c, and C-c, represent statistically significant, denoting *** *p*  <  0.0001.

To analyze the effects of the variants on NBD1 conformational stability, we quantified the radius of gyration (Rg) of all trajectories, which indicates the changes in the overall shape of the protein structure due to the amino acid substitution [51]. As shown in Figure 3(Aa), the Rg plot shows a distinct pattern among all systems, where G1050V shows notable fluctuations compared to the others. Remarkably, after 150 ns, the Rg values of the wild-type structure remained stable over time, which was ~20 Å. On the other hand, S1067C showed higher Rg value compared to those of the wild-type, although the curve shows a stable trend along the time. Rg distribution functions of wild-type, G1050V, and S1067C variants were plotted in Figure 3(Ab), which shows slightly overlapped in the occurrence frequencies of the Rg in all systems. The Rg distribution of S1067C was seen to shifted to the right compared to wild-type and showed a high distribution that occurred in the range of 20.1 Å to 20.3 Å. The G1050V showed a more extensive distribution along with lower density frequencies compared to the other systems. Overall, the results indicate that G1050V significantly induces compactness than the wild-type, while S1067C induces flexibilities (Figure 3(Ac)). The conformational stability as a mean of the intramolecular hydrogen bond was evaluated and plotted concerning the time (Figure 3(Ba)). Although all systems show no distinctive pattern in the total number of H-bonds over the time, G1050V resulted in the lower distribution (shifted to the left), while the H-bond distribution in wild-type and S1067C were entirely overlapped with each other (Figure 3(Bb)). Similarly, in SASA calculation, which denotes solvent accessibility [45], the variants decrease the solvent accessibility (Figure 3(Cb,c)) than the wild-type, indicating a decrease in protein folding due to the variants. 

#### 2.4.2. Effects of Variants on Protein Dynamics

**Figure 4:**
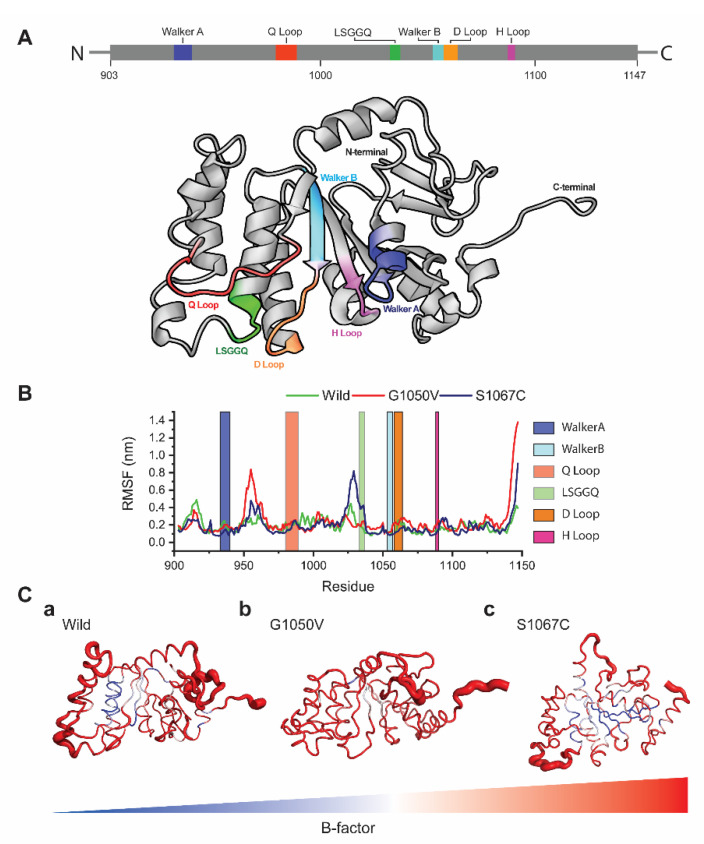
Effects of variants in residual fluctuations. (**A**) Structural representation of the NBD1 domain highlighting the different signature motifs involving in dimeric nucleotide-binding, ATP hydrolysis and TMDs conformational change. (**B**) The root mean square fluctuation (RMSF) plots for both wild and variants types, where green, red, and dark blue line describes wild, G1050V, and S1067C, respectively. (**C**) (**a**–**c**) Tube representation of the NBD1 domain highlighting the B-factor calculated from the RMSF values. The broader tube with red shades represents the area with high B-factors, while a narrower tube with blue shades indicates residues having low B-factor.

To analyze the effects of variants on the dynamic behavior of the NBD1 domain, we first calculated RMSF values of each residue using resulted trajectories, which indicates the degree of atomic displacement [52,53]. The NBD1 domain of ABCA1 consists of six major signature motifs, including Walker A and B, LSGGQ, and Q/D/H loops. The RMSF graph shows that variants caused major changes in the residual fluctuations compared to the wild-type, in which G1050V shows the highest fluctuations in the regions of 950–968 and the H loop (residues, 1087–1090), respectively (Figure 4B). Comparatively, S1067C induces the highest fluctuation in the LSGGQ loop and reduced flexibility in both Walker A and Walker B motifs. Cumulatively, all variants caused high fluctuations in the C terminal region compared to wild-type (Figure 4C). 

**Figure 5:**
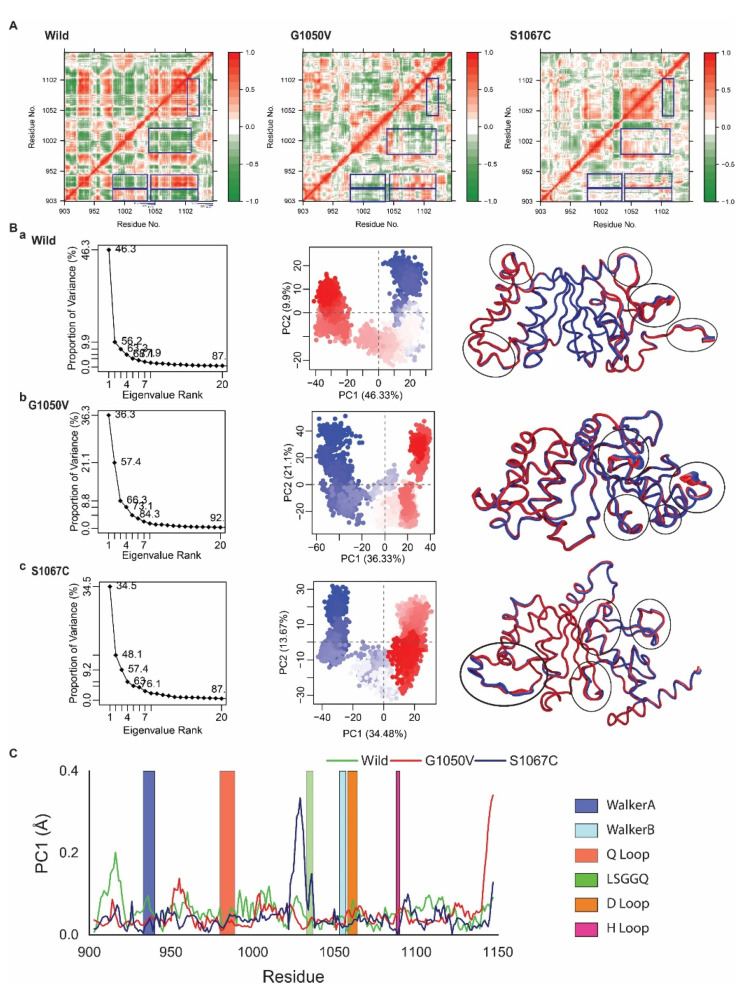
Effects of variants on protein dynamics movement. (**A**) Representative correlated dynamic cross-correlation maps (DCCMs) generated from simulated trajectories of wild-type, G1050V, and S1067C. Here the residue-residue cross-correlations are visualized in a two-dimensional heatmap with a colored codded representation of green to white to red. The residual movement that is correlated is represented as a red color, while green color indicates a negative correlation, and white means random motions. (**B**) Principle Component Analysis (PCA) on the simulated trajectories of wild-type, G1050V, and S1067C. Here, the projection of simulated trajectories of an individual system, including wild-type (**a**), G1050V (**b**), and S1067C (**c**), is represented in the left panel, where the middle section shows the conformational distribution of each protein captured on in the first two principal components. Here, each dot describes the protein conformation, where simulation time is represented by a color-coded scale from blue to white to red. The right panel shows total atomic displacements of the protein for PCA1, which is visualized in the tube model, indicating protein flexible region by broader tube. (**C**) Residue-wise mobility plot for PC1, where green, red, and dark blue describe wild, G1050V, and S1067C, respectively.

We next analyzed Dynamic Cross-Correlation Map (DCCM) analysis, which addresses correlated and anticorrelated motions of the protein that occurred during the simulation [52]. The results from DCCM analysis are represented in a color-coded heatmap, as shown in Figure 5A, where red color highlights positively correlated motions between the specific residues, while blue indicates negatively correlated motions. The results revealed substantial differences in both correlated and anticorrelated motions between the wild-type and variants. As represented in the DCCM of the wild-type, the residues 903 to 925 showed positive correlated motion with the residues 983–1039; however, they showed anticorrelations with the residues 1043 to 1127. Furthermore, residues containing Walker A motif covered in 928 to 940 showed anticorrelation with 983–1039, which contains both Q loop (980 to 989) and LSGGQ motif (1033 to 1037), respectively. Besides, residues present in Walker A motif also showed a positive correlation with the residues 1043 to 1127, containing H loop (1088 to 1090). Moreover, residues 983 to 1022, including the Q loop, also maintains a negative correlation with the residues 1037 to 1115, which included H loop (1088 to 1090). An additional positive correlation was also observed between the residues 1040 to 1105 and 1102 to 1130. However, these correlated motions were seen to change in the variants, where most of the motions that occurred in the regions (highlighted in the blue box) were changed into either random or anticorrelated manner. Specifically, S1067C reduced both correlated and anticorrelated motions that occurred in 903 to 925 (with 983 to 1039; 1043 to 1127) in wild-type. S1067C also changed correlated motion into anticorrelated, which present in the region of 1040 to 1105 (correlated with residues, 1102 to 1130). However, G1050V reversed the motions compared with wild-type, which occurred in the regions including 983 to 1039 and 1043 to 1127, maintaining relation with residue 903 to 925. Moreover, a negative correlation between 983 to 1022 and 1037 to 1115 showed in wild-type was slightly reduced by G1050V but completely removed by mutant S1067C.

In order to gain further insights, we next performed Principle Component Analysis (PCA), which represents the most active motion of the atom by means of eigenvalues and eigenvectors. The PCA is calculated based on the construction of the diagonal covariance matrix from Cα atom of the protein, where the atomic motion is represented by eigenvectors, while eigenvalue describes atomic contribution in the motion [54]. PCA calculated for wild and variants are represented in Figure 5B, where the first three PCs in wild, G1050V, and S1067C were demonstrated to have 46.33%, 36.33%, and 34.48% of the total variance in the motion, respectively (Figure 5B, shown in the left panel). It was also noticed that the score for the total variance in wild structure for the first PC was 87%. Similarly, for G1050V and S1067C, the scores were 92% and 87%, respectively, indicating that G1050V increased the motion of the protein. Again, Figure 5B (middle panel) displayed two conformational distributions by red and blue dots where the white dots represented intermediate states. The continuous color scale from red to white and white to blue highlights periodic jumps among different conformations. In the wild-type, the majority of the blue and red dots were dispersedly distributed in a scattered state, as shown in Figure 5(Ba), while the majority of the red and blue dots were convened in G1050V, and most of the conformations were distributed separately around the right and left sight (Figure 5(Bb)). Therefore, it could be suggested that the wild protein was quite flexible during the simulation than the mutant G1050V.

Moreover, in Figure 5(Bc), most of the blue and red scattered dots in S1067C were assembled well and also distributed around the left and right side as G1050V, indicating most of the conformations in S1067C was stable as G1050V. However, S1067C showed less transition state than G1050V, which recommended that the mutant S1067C could make a more stable system than G1050V. From Figure 5C, local residue mobility in terms of PC1 was shown to understand the conformational changes of wild protein in which maximum changes were increased in the active site loops though it was reduced in the variants. S1067C increased the movement in the LSGGQ loop but highly decreased in the H loop compared to wild protein. In G1050V, it also decreased the movement in the Q loop, LSGGQ loop, while increased in the H loop compared to both wild and S1067C variants.

#### 2.4.3. Effect of Variants on Intra-Protein Communication

**Figure 6:**
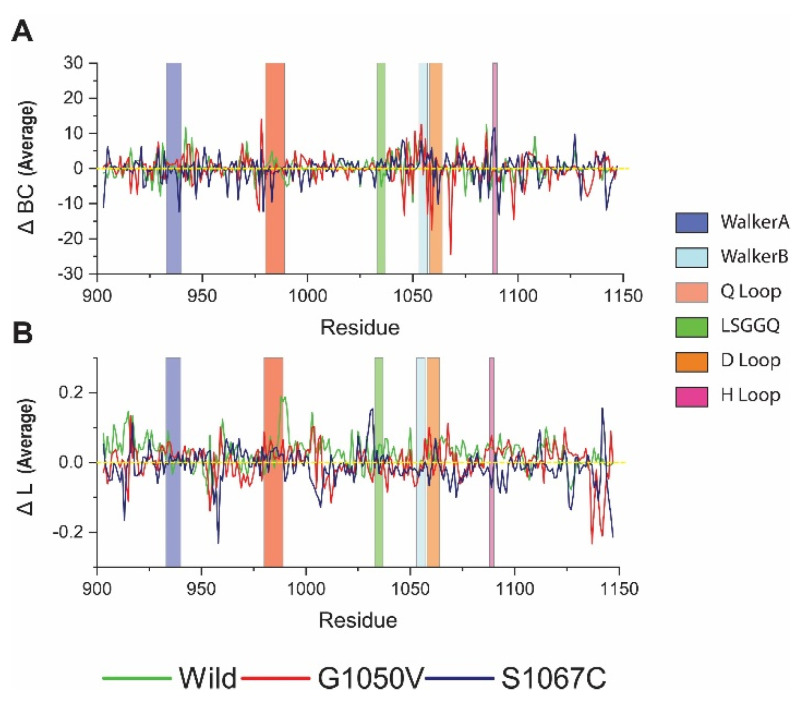
Analysis of the effect of variants in dynamic residue network. (**A**) Betweenness centrality (ΔBC); (**B**) average shortest path (ΔL). Here, green, red, and dark blue describe wild, G1050V, and S1067C, respectively.

Since the presence of variants causes a substantial effect in the NBD1, as evidenced from the above analysis, to analyze more closely, we used a graph theory technique to describe the effect of variants in residual communication, which is directly correlated with protein function [55,56,57,58]. The results are presented as average betweenness centrality (BC) and average shortest path (L), where BC indicates major residue involved in inter-domain communication, while L denotes residue reachability [57,58]. Average ΔBC analysis showed that the residue 942, 1085, and 1108 are essential for communication, as they obtained high value in the wild-type (Figure 6A). On the other hand, variants showed a reduction of BC to these residues. Furthermore, all variants showed a considerable reduction of ΔBC in the Q loop than the wild-type. Comparatively, G1050V reduced ΔBC in the residues, including 977, 1046, 1057, 1059, 1068, and 1098. Conversely, S1067C reduced ΔBC of overall residues, which are located in Walker A and B and also in the D loop. Furthermore, we calculated residual usage by identifying the ΔBC difference between wild and variants. As shown in Figure S2 (Supplementary File 3), S1067C reduced overall residue usage, although it increased in the H loop. On the other hand, G1050V reduced residue usage in the Walker B and D loop, which is around the site of mutation.

Figure 6B shows the average shortest path of the residue in the protein, in which high value represents the responsible residue steering protein conformational change [55]. Interestingly, maximum residues in wild-type protein represent the highest ΔL values, which describes the dynamics behavior of the protein [55]. However, variant reduces ΔL values in overall residues of the protein, suggesting a reduction of protein flexibility. Furthermore, G1050V induced ΔL values in Walker A and D loop, particularly residue 1058, and increased in Walker A motif, including residues 935 to 937. Conversely, S1067C increased ΔL values only in residue 1031 to 1032 and reduced in most of the residues of the protein, which suggests protein compactness, is consistent with Rg analysis. 

#### 2.4.4. Effect of Variants in Protein Secondary Structure Elements

**Figure 7:**
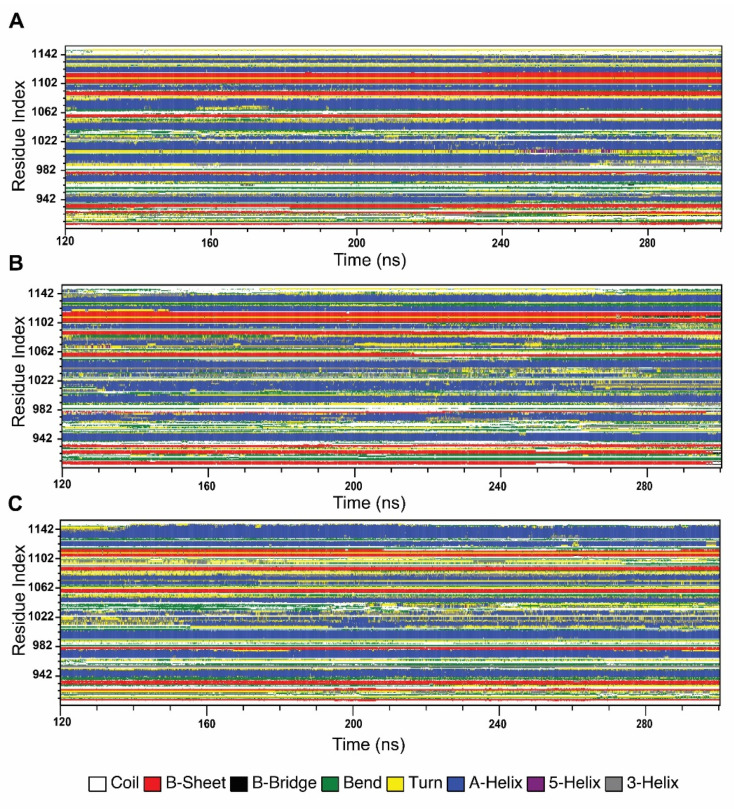
Effect of variants in the stability of the secondary structure elements of the NBD1 domain during the simulation. (**A**) Wild-type, (**B**) G1050V, (**C**) S1067C.

In order to understand the changes in protein secondary structural arrangement made by variants, we analyzed secondary structure elements of both wild-type and variant types, using Define Secondary Structure of Proteins (DSSP) algorithm, shown in Figure 7. SSE analysis shows that G1050V introduced more β-strand conformation in the residues ranging from 903 to 940, specifically in the region of 903 to 913 and 922 to 927, whereas it disrupted β-strand in Walker A motif, residues 933 to 940 while sustaining A-helix formation in the residues 942 to 950. The region in LSGGQ, residues 1033 to 1037, was also seen to be formed by A-helix conformation in G1050V. Furthermore, G1050V inhibited A-helix formation in the residues ranging from 1065 to 1085 by causing a bend and turn conformation while it decreased β-strand in the Walker B motif (residues, 1053 to 1057). In addition, G1050V stabled more A-helix formation in the region 1025 to 1040, while this region showed time-dependent coil, bend, and turn conformation in the wild-type. 

On the other hand, S1067C interrupted A-helix formation in Walker A motif, residues 935 to 940, by introducing bend conformation. Interestingly, S1067C caused major conformational changes in the LSGGQ motif, where it turned A-helix conformation to bend and coil. As a result, this region shows high mobility and flexibility, which is consistent with RMSF, PCA, and DCCM analysis. Furthermore, S1067C enhanced bend conformation in the residues 1053 to 1064, which contains Walker B and D loop motifs. In addition to that, S1067C caused a significant alteration in the c-terminal region; especially, it induced bending conformation in 1092 to 1105 by inhibiting A-helix conformation while reducing β-strand in 1105 to 1113 and increasing A-helix conformation in the regions 1115 to 1146. 

#### 2.4.5. Free Energy Landscapes (FELs)

**Figure 8:**
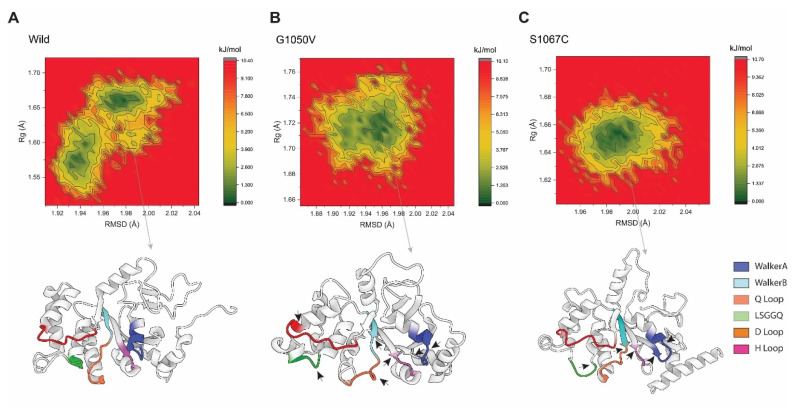
Free energy landscape analysis representing the conformational dynamics of the protein in (**A**) wild-type, (**B**) G1050V, and (**C**) S1067C, during the simulation. Here, the counter map was constructed based on the RMSD and Rg of each protein as a reaction coordinate highlighted as the X and Y-axis. The color scheme to the right of each plot is given in kJ/mol, where deep green represents protein conformation with a low energy state, while red describes a high energy state. The bottom of each plot shows the representative structural snapshot present in the stable minima, where the arrowhead marks the conformational change.

In order to understand the conformational difference between the wild-type and variants, FEL was performed, which was constructed based on the reaction coordinates of RMSD and the radius of gyration (Rg), respectively. As shown in Figure 8, the energy minima basins are represented from red to green, where concentrated green zones indicate more stable conformation with minimum energy and less stable conformation is denoted by red zone having the highest energy conformational state. The shape and size of the area with minimal energy represent the conformational stability of the protein [59]. As represents in Figure 8, the basin in the wild-type splits into two energy minima, although it is concentrated and centralized, representing the stability of the protein, and it was flexible during the simulation. On the other hand, the variants showed single energy minima but were less concentrated than the wild-type. Although the basin in G1050V was seen to be more dispersed than the S1067C, the basins with low energy minimum were more concentrated than S1067C, which was around Rg values between 1.70 Å and 1.73 Å and RMSD between 1.94 Å and 1.97 Å, respectively. This observation suggests that the conformational dynamics were less G1050V than the S1067C, which agrees with Rg analysis. The stable conformation with the least minimum free energy was extracted for each simulation system, which shows a substantial difference not only in the active site of NBD1 but also in the overall protein conformation (as shown by a black arrowhead in Figure 8B,C). 

## 3. Discussion

Accumulating data from a large number of studies so far suggested the role of several mutations in the progression of various diseases; however, not much is known about the role of ABCA1 variations in relation to natural polymorphisms. It is, therefore, crucial to identify deleterious nsSNPs in the *ABCA1* gene, as nsSNPs cause the most significant damaging impact on the protein structure and function [60]. The current study only considered missense variants because of their direct involvement in disease pathologies and their impacts on the adopted treatment regimen [61]. To analyze the effect of a large number of nsSNPs, using bioinformatics tools is a cost-efficient option, which allows identification of damaging SNPs with functional importance and with detailed analysis of an individual’s disease susceptibility followed by the investigation of the structural basis of disease-causing mutations [62,63,64]. To date, many computational tools have been developed to identify highly damaging nsSNPs; however, the computational approach with a combination of multiple algorithms, consisting of both sequence-based and structure-based methods, provides a means of more reliable and accurate prediction [43,65]. In the present study, we retrieved 1421 missense SNPs that we thus considered in a total of nine in silico deleterious predictors, where SIFT, PANTHER, PROVEAN, SNP & GO, I-Mutant 3.0, and PhD SNP use sequence-based information; in contrast, PolyPhen 2, and PredictSNP use mixed/consensus information (sequence and structure) to predict the disease-associated or deleterious SNPs. We observed that S741C, Y793C, S1067C, and G1050V were the most deleterious SNPs, supported by at least seven out of nine tools, concluding that the results obtained can be reliable, as the respective tools run on different algorithms to provide a similar pattern of results [66]. 

It should be noted that the *ABCA1* gene serves high conservancy between the species, where ABCA1 in human shares 95.2% identity with the mouse, while it is 85.3%, 25.5%, and 21.6% similar to the chicken, drosophila, and *C. elegans*, respectively [67,68]. Hence, the degree of conservancy was further analyzed with the help of the ConSurf server, which classified G1050V and S1067C as functional and highly conserved. The molecular dynamics simulation characterized both nsSNPs in order to gain further insight into the conformational changes causing variants. The results showed that nsSNPs significantly impacted overall protein conformation, citing an example of G1050V that makes the protein structure rigid, while S1067C induced protein flexibility, as revealed by Rg and SASA analysis.

As highlighted in Figure 4A, the six major signature motifs in ABCA1, including Walker A and B, LSGGQ, and Q/D/H loops in the NBD1 domain, are responsible for the ATP binding, ATP hydrolysis, NBD-NBD communication as well as in the conformational changes of TMDs. The conformational changes in TMDs is a primary requirement for substrate transportation [69]. During the simulation, conformational stabilities of all of these motifs are found to be altered by variants. The Walker A and B motifs, consisting of the residues ranging from 933 to 940 and 1053 to 1057, respectively, significantly contribute to the binding of ATP. Residues in these motifs directly interact with γ-phosphate moiety and ribose ring of the ATP through hydrogen bonding [70]. 

The results from the MD simulation showed that G1050V induced random motion in both Walker A and B motifs (Figure 5) and reduced principle dynamic motion (Figure 5C); as a corollary, more coil and bending conformations were introduced in these regions (Figure 7B), indicating significant structural alternation. On the other hand, S1067C induced more random to positively correlated motion in these motifs, and by doing so, S1067C markedly reduced dynamics motion and introduced more turn and bend conformation in these regions (Figure 7C). 

The LSGGQ, also known as C motif, is highly conserved among the ABC transporters and maintained essential functions in these proteins by maintaining residual communication with another NBD domain (NBD2) and also involved in phosphate binding [71]. The previous report suggested that mutation in this region resulted in the loss of ATP binding, which is a result of impairing protein function through disrupting inter-domain communication [72,73]. Both G1050V and S1067C changed the correlative motion in LSGGQ motif in the simulation, where S1067C caused the highest fluctuation in this segment by introducing bend and turn conformation, while G1050V caused the formation of more A-helix conformation in this region. Loo et al. suggested that the conformational flexibility of the LSGGQ motif is necessary for ATP binding, which was found to be disrupted by both G1050V and S1067C in the present study [74]. It has been ascertained that amino acid substitution with different size and polarity scales disturbs the internal residue network and contributes to the loss of native inter-residue interaction, thus affecting protein stability, which provides a possible mechanism of deleteriousness [75]. Complying with this concept, in G1050V, substitution with valine, which is more hydrophobic and relatively larger than glycine, causes the change in the residual communication, which mainly affects the Walker B motif and D loop. Unlike G1050V, the replacement of nonpolar cysteine in C1067S with polar serine steers conformational change by changing residual networks in both Walker motifs and D loop, although both residues are similar in size. Along with this information, the cumulative findings from MD simulation demonstrate that nsSNPs caused a substantial impact on the active site of ABCA1 and thus might contribute to the loss of ATP binding, ATP hydrolysis, and the alteration of TMDs conformational changes, supporting the pathogenic behaviors of nsSNPs in the ABCA1 function. 

The association between altered HDL concentrations and many other ABCA1 variants, including R219K (rs2230806) is well established by previous studies [76,77,78], which are linked to the development of ischemic heart disease (IHD) by the increasing risk of atherosclerosis in the general population [79]. According to recent studies, it has been revealed that the effects on plasma HDL cholesterol level and the incidence and severity of coronary heart diseases depend mostly on the distribution and frequency of SNPs in different regions of the *ABCA1* gene as well as different populations [80,81]. Since similar SNPs show similar or opposite effects, widespread mutagenesis studies are required to investigate the exact role and the loss of function of ABCA1 upon G1050V and S1067C mutations. Besides, targeting ABCA1 for upregulating plasma HDL level in the normal population has now been a recognized therapeutic strategy [82]; however, patients with defective ABCA1 variants are recommended to be treated with the HDL-enhancing drugs other than targeting ABCA1 [80,83]. Therefore, understanding the role of ABCA1 variants (G1050V and S1067C) in HDL regulation and drug response would provide clinical and biochemical presentations for selecting appropriate drug candidates. 

However, tools used in this study to screen out deleterious SNPs generally depend on the machine learning algorithms, and the mutations present in the highly conserved area do not always produce noticeable phenotypes. Thus, magnitudes of the damaging effects of these identified SNPs should be validated by establishing a genotype/phenotype correlation-based study. To our knowledge, there is no clinical evidence relating to these identified variants at any population that needs exploration to validate this investigation. Overall, this study offers a starting point to investigate the structural influence in designing rational drugs, considering the deleterious variants. The prevalence of these mutations among different geographical locations also needs further investigation.

## 4. Materials and Methods 

### 4.1. Data Retrieval and In Silico Deleterious nsSNP Prediction

The relevant *ABCA1* gene variation datasets were retrieved from the NCBI SNP database with their corresponding rsID. The damaging effect of retrieved variants was characterized by employing several in silico bioinformatics tools [84]. The nine most widely accepted tools were used, which are aimed to recognize only damaging SNPs. Tools including sorting intolerant from tolerant (SIFT) [85], polymorphism phenotyping v2 (PolyPhen-2) [86], protein variation effect analyzer (PROVEAN) [87], I-Mutant 3.0 [88], PredictSNP [89], PANTHER [90], single nucleotide polymorphisms and gene ontology (SNP & GO) [91], predictor of human deleterious single nucleotide polymorphisms (PhD SNP) [92], and multivariate analysis of protein polymorphism (MAPP) [93] were used in this study. Here, the SIFT is a multi-step algorithm that predicts deleterious mutations based on the sequence homology approaches. Moreover, SIFT uses the PSI-BLAST algorithm [45]. Similarly, PolyPhen-2 uses the position-specific independent counts (PSIC) scoring method utilizing the Bayesian classifier. In polyphen-2, two models were used; HumDiv and HumVar, whereas HumVar identifies the extreme phenotypes, and HumDiv classifies less damaging SNPs using PSIC [94]. PROVEAN use alignment-based clustering and sequence clustering score to predict the alteration of the biological function of a protein due to mutation [95]. I-Mutant 3.0 uses a support vector machine (SVM) algorithm to calculate the stability of the mutant protein structure by the change of Gibbs-free energy (DDG). However, the DDG value estimates the direction in which the mutation transfers the stability of the protein, rather than directly predicting the comparative stability modifications regarding mutation [45]. The latest tool PredictSNP, used for classifying disease-associated SNPs from the neutral ones, which accumulate various computational tools and provide results generated from all these tools, which is much convenient and reliable. PANTHER also uses SVM algorithm and evolutionary relationships to find out deleterious SNPs. SNP & GO provides the probability values of disease-associated mutation using gene ontology (GO) database based on molecular and functional information [91]. PhD SNP uses SVM method to classify disease-associated mutation from neutral SNPs using the UniRef90 database and BLAST algorithm [96]. 

### 4.2. Conservation Analysis

Consurf is a computational tool that uses the phylogenetic relationship within the homologous sequences and analyzes amino acid conservation in a protein or nucleotide in RNA or DNA [97]. In this study, the conservation analysis was calculated from the protein sequence based on the Bayesian calculation method [98,99]. A score from 1 to 4 is considered as a variable, whereas a score between 5 and 6 and 7 to 9 was considered as intermediate and conserved, respectively [53].

### 4.3. Molecular Dynamic Simulation

#### 4.3.1. Preparation of Simulation System

We considered the structure of NBD1 domain in wild-type and variants (G1060V and S1067C) for MD simulation in order to get detailed insights on the deleterious effect of nsSNPs. Accordingly, the NBD1 domain was retrieved from the three-dimensional crystal structure of ABCA1, which was obtained from the protein databank, bearing id of 5XJY [50]. We prepare the retrieved structure for simulation by removing water molecules, adding hydrogen bonds and fixing bond orders and charges at a neutral pH. Besides, the structure was corrected by adjusting the amide groups in asparagines and the protonation states of histidines, aspartic acids, and glutamic acids. Energy minimization was applied to correct the root mean square deviation (RMSD) of the heavy atom in protein structure down to 0.30 Å, which was done using the optimized potentials for liquid simulation (OLPS3) force field. The variant structures, G1060V and S1067C, were constructed using a script of computational mutagenesis, mutant residue, which is embedded in Schrödinger suite 2017-1 software (Schrödinger, LLC, New York, NY, USA) [100,101]. After that, a short MD simulation (for 500 ps) was performed to refine the resultant structures, which includes YAMBER3 force field [102]. The simulation was done in a solvent system with a density of 0.997 and a pH and temperature of 7.4 and 298 K, respectively. The resultant structure with the lowest energy was selected for further analysis. 

YASARA Dynamics software (YASARA Biosciences GmBH, Vienna, Austria) was used for MD simulation, where each protein structure was parameterized with the AMBER14 force field [103,104], after optimizing the hydrogen bond network. We used transferable intermolecular potential3 points (TIP3P) water model to solvate the protein in a cubic simulation cell with a periodic boundary condition by maintaining the solvent density of 0.997 gL^−1^ [105]. During solvation, the acid dissociation constant value (pKa) was calculated for the amino acid present in the protein. Also, SCWRL algorithm, combined with the H-bonding network optimization, was applied to maintain the correct protonation state of each amino acid [106]. The physiological condition in the simulation was maintained by setting pH at 7.4, with the addition of Na^+^ and Cl^−^ ions [107]. The energy of each simulation system was minimized using the steepest gradient approach (5000 cycles), which follows a simulated annealing method. With a multiple time-step algorithm, the timestep interval in all simulations was set to 2.00 [108,109]. The Ewald particle mesh (PME) method was applied to describe the long-range electrostatic interactions with a distance cut-off was set to 8 Å [110]. Following Berendsen thermostat and constant pressure, the simulation was conducted for 300 ns, from which the simulated trajectory was obtained with a time step interval of 25 ps. Furthermore, the trajectories of data from simulation were analyzed by the default script of the YASARA [111] macro, and VMD software (Version 1.9.3, Theoretical and Computational Biophysics Group, Urbana, IL, USA, 2016) [112] was used to determine RMSD, RMSF (RMS fluctuations) [53], and secondary structures by DSSP (EMBL, Heidelberg, Germany) [113,114] tools. 

#### 4.3.2. Dynamic Cross-Correlation Map (DCCM) and Principle Component Analysis (PCA)

DCCMs were implemented to show the inner dynamics of protein conformations throughout the simulation. Here, The Bio3D [115] package integrated with R program was used. Bio3D DCCM provides Pearson’s co-variance matrices correlation coefficients, calling on “cov2dccm” and the following equation can be used to calculate it:

$$C_{ij}=\frac{\langle ∆r_{i}.∆r_{j}\rangle }{{\left\{{\langle ∆r_{i}\rangle }^{2}{\langle ∆r_{i}\rangle }^{2}\right\}}^{1/2}}$$

where a cross-correlation ratio, C_ij_, has been considered for the Cα electrons [116]. ∆r_i_ and ∆r_j_ represent the average location of ith and jth residues, respectively, and the angular brackets symbolize the time average. The calculated values in DCCM ranged from −1 to +1, where positive values represent positive correlation and negative values represent the anticorrelation. Similar software was also used for PCA analysis, which calculates the dynamics of a protein in any biological system. The details of the PCA have been previously described [52].

#### 4.3.3. Dynamic Residue Network Analysis

Molecular dynamics task (MD-TASK) was used for residue network analysis, which allows the user to study inter and intradomain communication by using graph theory, where protein is known as a residue interaction network (RIN), counting each residue node in the network in the protein network [55]. In the network, the residue interaction was constructed based on the pairwise distances (with a cut off 6.7 Å) between Cβ atoms (Cα for glycine) of all residues present in the protein [117]. We considered all frames of the MD trajectory. 

In this study, dynamic residue network analysis (DRN) was calculated using MD-TASK with respect to the changes in betweenness centrality (BC) of each residue and average shortest path length (*L*) over the trajectory. 

The importance of a residue in protein dynamics communication was calculated by the BC, which is equal to the number of shortest paths from all nodes to all others that pass through that node. On the other hand, the average shortage path (L) for each residue in the protein is calculated by measuring the total of shortest paths of each residue to the other residues of the protein, which is divided by the total number of residues minus one. Betweenness centrality and shortest path length were calculated by using calc_network.py and avg_network.py scripts. The mathematical details of the tools have been previously described [118].

#### 4.3.4. Free Energy Landscape (FEL) Analysis

FEL was obtained using a conformational sampling method providing all the probable macromolecular structural conformation [119]. The stability of protein was determined by calculating Gibb’s free energy in the analysis of FEL. In this study, FEL was calculated by the following equation:

$$G_{i}=-K_{B}Tln\left(\frac{N_{i}}{N_{max}}\right)$$

where G_i_ is the Gibbs free energy of state, kB. T stands for temperature (which was set at 300 K). N_i_ and N_max_ determine the population bin I and the most inhabited population bin, respectively. As the smallest provability, an unnatural barrier scale was set for the bin without any populations. A color-code model presented different levels of energy.

### 4.4. Statistical Analysis

For predicting correlation among various bioinformatics tools, SPSS v19 software was used. The *t*-test and single-factor ANOVA test were used for comparison to find the most significant combinations. Statistical analysis for MD trajectories was performed in GraphPad Prism v 8.0 (GraphPad Software, San Diego, CA, USA) software. *p* values of <0.0001 were deemed to be highly significant. 

## 5. Conclusions

Genetic variants in ABCA1 are associated with chronic and age-associated disorders; therefore, it is necessary to unveil the deleterious nsSNPs in *ABCA1* and their damaging effects in ABCA1 loss of function. Using several in silico deleterious SNP predictors, the present study identified G1050V and S1067C as two potential nsSNPs, located in the NBD1 domain of ABCA1. Both of the nsSNPs were found to disrupt the overall conformational dynamics of the NBD1 domain. Furthermore, the variants also disrupted the plasticity of the active site by causing significant structural alterations in the ATP binding motifs, which might be responsible for the loss of ATP binding. Hence, these SNPs can be considered as important candidates for analyzing their phenotype occurrence in the diseases associated with ABCA1 loss of function. 

## Figures and Tables

**Table 1:** Cumulative analysis of most deleterious variants in ATP-binding cassette transporter A1 (ABCA1).

rs ID	Substitution	SIFT	PolyPhen 2		MAPP	PANTHER	SNP & Go	PhD-SNP	PredictSNP	PROVEAN	I-Mutant 3
			HumDiv	HumVar							
rs762112742	S741C	0.05 *	1 *	0.996 *	-	0.663 *	0.761 *	0.803 *	D *	−4.265 *	−1.01 *
rs1461682152	Y793C	0.05 *	0.994 *	0.964 *	-	0.654 *	-	0.607 *	D *	−6.46 *	−0.86 *
rs985622413	G1050V	0 *	1 *	0.999 *	-	0.568 *	-	0.733 *	D *	−6.192 *	−0.58 *
rs1394779021	S1067C	0 *	0.999 *	0.954 *	-	0.671 *	-	0.858 *	D *	−3.494 *	−0.58 *

The listed four nsSNPs are predicted as damaging or deleterious or effect and agreed commonly to by SIFT, Polyphen-2 HumDiv, Polyphen-2 HumVar, PROVEAN, I-Mutant 3.0, PredictSNP, PhD-SNP, SNP & GO, PANTHER, and MAPP tools, where * means deleterious.

## Supplementary Materials

The following are available online at https://www.mdpi.com/1422-0067/21/20/7606/s1, Figure S1: Analysis of Root-mean-square deviation (RMSD) based on the Cα atoms for wild-type and NBD1 variants, which were calculated by comparing the starting structure of each simulation, Figure S2: Effect of variants in the change of (A) average betweenness centrality and (B) average shortest path (variant minus wild).
