# Supplementary material for: Computational SNP Analysis and Molecular Simulation Revealed the Most Deleterious Missense Variants in the NBD1 Domain of Human ABCA1 Transporter
Source: Int J Mol Sci. 2020 Oct 14;21(20):7606. doi: 10.3390/ijms21207606 (PMC7589834; doi:10.3390/ijms21207606)
Supplement: Supplementary file 1 [file ijms-21-07606-s001.zip › Supplementary Files/Supplementary File 2.pdf]

ConSurf Results

```
1
M A C W P Q L R L L
e e e e e e b b b b
f         f     s s

11
L W K N L T F R R R
b b e e b e e e e e
s s f f   f   f f f

21
Q T C Q L L L E V A
e e b e b b b e b b
f     f       f

31
W P L F I F L I L I
b b b b b b b b b b
                s

41
S V R L S Y P P Y E
b b e e e b e e e e
  s f         f

51
Q H E C H F P N K A
e e e b e b e e e e
        f s f f f f

61
M P S A G T L P W V
b e e e e b b b b b
s f f   f     s s

71
Q G I I C N A N N P
e b b b b e b e e e
f   s   s f     f f

81
C F R Y P T P G E A
b e e e e e e e e e
s         f   f f

91
P G V V G N F N K S
e e e b b b b e e b
f f       s s     s

101
I V A R L F S D A R
b b b e b b e e b e

111
R L L L Y S Q K D T
e b b e e e e e e e

121
S M K D M R K V L R
e b e e b e e e b e

131
T L Q Q I K K S S S
e b e e b e e e e e

141
N L K L Q D F L V D
e b e b e e b b e e

151
N E T F S G F L Y H
e e e b b e b b e e
  f           s

161
N L S L P K S T V D
e b e b e e e b b e

171
K M L R A D V I L H
e b b e b e b e b e

181
K V F L Q G Y Q L H
e b b e e b e e b e

191
L T S L C N G S K S
b e e b b e e e e b

201
E E M I Q L G D Q E
e e b b e b e e e e

211
V S E L C G L P R E
b b e b b e b e e e
        s

221
K L A A A E R V L R
e b e e b e e e b e

231
S N M D I L K P I L
e e b e b b e b b e

241
R T L N S T S P F P
e e b e e e e e e e

251
S K E L A E A T K T
b e e b b e b b e e

261
L L H S L G T L A Q
b b e e b e e b b e

271
E L F S M R S W S D
e b e e e e e b e e
            f

281
M R Q E V M F L T N
b e e e b e b b b e

291
V N S S S S S T Q I
e e e e e e e e e b

301
Y Q A V S R I V C G
b e b b b b b b b b
                  s

311
H P E G G G L K I K
e e e e e e e e b e

321
S L N W Y E D N N Y
b b e b e e e e e b
s   f     f f

331
K A L F G G N G T E
e b b b e e e e e e
        f

341
E D A E T F Y D N S
e e e e e e e e e e

351
T T P Y C N D L M K
e e e e b e e b b e
        s

361
N L E S S P L S R I
e b e e e e b b e b
    f     f

371
I W K A L K P L L V
b b e b b e e b b b
  s       f f

381
G K I L Y T P D T P
b e b b b b e e e e
s f         f   f f

391
A T R Q V M A E V N
b b e e b b e e b e
              f   f

401
K T F Q E L A V F H
e e b e e b e e b e
  f s

411
D L E G M W E E L S
e b e e b b e e b e
          s   f

421
P K I W T F M E N S
e e b b e b b e e b
f

431
Q E M D L V R M L L
e e b e b b e e b b
            f

441
D S R D N D H F W E
e e e e b e e b b e

451
Q Q L D G L D W T A
e e b e e e e b e b

461
Q D I V A F L A K H
e e b b e b b e e e
          s

471
P E D V Q S S N G S
e e e e e e e e e e

481
V Y T W R E A F N E
e b e b e e b b e e
      s

491
T N Q A I R T I S R
b e e b b e b b e e

501
F M E C V N L N K L
b b e b b e b e e b
      s         f

511
E P I A T E V W L I
e e e e e e e e b b
          f

521
N K S M E L L D E R
e e b b e b b e e e
    s       s

531
K F W A G I V F T G
e b b b b b b b b b
    s s       s

541
I T P G S I E L P H
b e e e e e e b e e
                f

551
H V K Y K I R M D I
e b e b e b e b b b
f       f s f s s

561
D N V E R T N K I K
e e b e e e e e b e
f   s f   f f f   f

571
D G Y W D P G P R A
e e e b b e e e e e
f     s   f f f f f

581
D P F E D M R Y V W
e e b e e b e b b b
  f     f   f s

591
G G F A Y L Q D V V
b b b b b b e e b b
s s s   s   f f
```

601
EQAIIRVLTG
eebbbebbee
f

611
TEKKTGVYMQ
eeeeeebbbe
     f   f

621
QMPYPCYVDD
ebeeebbbee
f fffs sff

631
IFLRVMSRSM
bbbbbbbebb

641
PLFMTLAWIY
ebbbbbbbbb
f   s

651
SVAVIIKGIV
bbbbbebbbb
 s s f s

661
YEKEARLKET
beeeeebeeb
 fff fsff

671
MRIMGLDNSI
bebbbbebbb
 f s

681
LWFSWFISSL
bbbbbbbebb
 s s   fs

691
IPLLVSAGLL
bbbbbbbbbb
     s

701
VVILKLGNLL
bbbbebbebb
      s s

711
PYSDPSVVFV
bbeeebbbbb
 sf

721
FLSVFAVVTI
bbbbbbbbbb
s   sss

731
LQCFLISTLF
bbbbbbbbbb
  ss s s

741
SRANLAAACG
bebebbbbbe
  sf s ssf

751
GIIYFTLYLP
bbbbbbbbbb
s s   sss

761
YVLCVAWQDY
bbbbbbbeee
s   s f

771
VGFTLKIFAS
beeebebbbe
     f   f

781
LLSPVAFGFG
bbebbbbbbb
  s ssss s

791
CEYFALFEEQ
bebbbebeee
sf   f fff

801
GIGVQWDNLF
ebebebeebe
f f fs f

811
ESPVEEDGFN
eeeeeeeebe
 ff   f

821
LTTSVSMMLF
bbbbbbbbbb
       s

831
DTFLYGVMTW
bbbbbbbbbb
s   s

841
YIEAVFPGQY
bbebbbebee
s f s fs

851
GIPRPWYFPC
bbeeeebbbb
s

861
TKSYWFGEES
eeeebeeeee

871
DEKSHPGSNQ
eeeeeeeeee

881
KRISEICMEE
eeeeeebeee
        f

891
EPTHLKLGVS
eeeebebbbb
 f   ss

901
IQNLVKVYRD
beebbebbee
   s f s

911
GMKVAVDGLA
eeebbbeebe
    ssf

921
LNFYEGQITS
bebbeeebbb
  s f fsss

931
FLGHNGAGKT
bebeeeeeee
 fsfffffff

941
TTMSILTGLF
eebbbbbbbb
ff s ss

951
PPTSGTAYIL
beeeeebbbe
sfffff

961
GKDIRSEMST
beebeeebee
s fs

971
IRQNLGVCPQ
beebbebbee
sf   f sff

981
HNVLFDMLTV
eebbbebbeb
 f ss sfs

991
EEHIWFYARL
eebbbbbbeb
ffs ss

1001
KGLSEKHVKA
eeeeeeebee
ff   s

1011
EMEQMALDVG
ebeebbeeee
f   f f

1021
LPSSKLKSKT
beeeeeeeee
s fff

1031
SQLSGGMQRK
eebeeebeee
  sfffsfff

1041
LSVALAFVGG
bbbbbbbbee
ssss sssff

1051
SKVVILDEPT
bebbbbbeee
 fss ssfff

1061
AGVDPYSRRG
eebbebbeeb
ffssf sff

1071
IWELLLKYRQ
bbebbbebee
ssfsssfsf

1081
GRTIILSTHH
eebbbbbbbb
ffsss ssss

1091
MDEADVLGDR
beebebbeee
sffsf sfff

1101
IAIISHGKLC
bbbbbebebb
 ssss s

1111
CVGSSLFLKN
beebebbbee
s fsf sf

1121
QLGTGYYLTL
eeeebbbbbb
  f s ss

1131
VKKDVESSLS
beeeeeeeee
sf

1141
SCRNSSSTVS
eeeeeeeebe

1151
YLKKEDSVSQ
eeeeeeeeee

1161
SSSDAGLGSD
eeeeeeeeee

1171
HESDTLTIDV
eeeeebebeb

1181
SAISNLIRKH
ebbbebbeee

1191
VSEARLVEDI
beebebbeeb
       f

```
1201
G H E L T Y V L P Y
e e e b b b b b e e
  f f           f

1211
E A A K E G A F V E
e e e e e e b b b e
    f

1221
L F H E I D D R L S
b b e e b e e e b e
          f

1231
D L G I S S Y G I S
e b b b b b b b b b
          s s     s

1241
E T T L E E I F L K
e b b b e e b b b e
f s   s f f

1251
V A E E S G V D A E
b b e e e e e e e e
s

1261
T S D G T L P A R R
e e e e e e e e e e

1271
N R R A F G D K Q S
e e e e e e e e e e

1281
C L R P F T E D D A
b e e e e e e e e e

1291
A D P N D S D I D P
e e e e e e e e e e

1301
E S R E T D L L S G
e e e e e e e e e e

1311
M D G K G S Y Q V K
e e e e e e e e b e

1321
G W K L T Q Q Q F V
e b e b e e e e b e
              f

1331
A L L W K R L L I A
b b b b e e b b e b
s s     f f

1341
R R S R K G F F A Q
e e e e e e b b b e
  f f     f     s f

1351
I V L P A V F V C I
b b b e b b b b b b
      f     s

1361
A L V F S L I V P P
b b b b b b b b b e
                  f

1371
F G K Y P S L E L Q
b e e e e e b e b b
        f

1381
P W M Y N E Q Y T F
e b b e e e e b e b
f     f     f     s

1391
V S N D A P E D T G
b e e e e e e e e e
  f   f

1401
T L E L L N A L T K
b e e b b e e b e e

1411
D P G F G T R C M E
e e e e e b e b e e
  f f         s

1421
G N P I P D T P C Q
e e e e e e e e b e
                s

1431
A G E E E W T T A P
e e e e e e e e e e

1441
V P Q T I M D L F Q
e e e e b e e b b e

1451
N G N W T M Q N P S
e e e b e e e e e e
                f f

1461
P A C Q C S S D K I
e e b e b e e e e e
f   s   s f

1471
K K M L P V C P P G
e e e b e e b e e e
    f   f   s f   f

1481
A G G L P P P Q R K
e e e e e e e e e e
f f f   f f f f

1491
Q N T A D I L Q D L
e e e e e b b e e b
                f

1501
T G R N I S D Y L V
e e e e b b e b b b
f   f f   s f

1511
K T Y V Q I I A K S
e b b b e b b e e e
f s

1521
L K N K I W V N E F
b e e e e b b e e b

1531
R Y G G F S L G V S
e e b b b b b b b e
f f s s   s   s

1541
N T Q A L P P S Q E
e e e b e e e e e e

1551
V N D A I K Q M K K
b e e b b e e b e e

1561
H L K L A K D S S A
e b e b e e e e e b

1571
D R F L N S L G R F
e e b b e e b e e b

1581
M T G L D T K N N V
b e e e e e e e e b
                f

1591
K V W F N N K G W H
e b b b e e e e b e
f   s   f f f f   f

1601
A I S S F L N V I N
b b b b b b b b b b
        s     s

1611
N A I L R A N L Q K
e b b b e b e b e e
f     s f     s

1621
G E N P S H Y G I T
e e e e e e b b b e
              s s

1631
A F N H P L N L T K
b b e b e b e b b e
    f s f s f s s f

1641
Q Q L S E V A L M T
e e b e e b b b b b
f f s f

1651
T S V D V L V S I C
b b b e b b b b b b
s s s f s   s s s

1661
V I F A M S F V P A
b b b b b b b b e b
s   s s s s s   f s

1671
S F V V F L I Q E R
b b b b b b b e e e
s s s s s s s f f f

1681
V S K A K H L Q F I
b e e e e e b e b b
s     f f f   f

1691
S G V K P V I Y W L
e e b e b b b b b b
f f s   s     s s

1701
S N F V W D M C N Y
b b b b b b b b e b
        s s     f s

1711
V V P A T L V I I I
b b b b b b b b b b
    s       s     s

1721
F I C F Q Q K S Y V
b b b b e e e e e e
s     s f f   f f f

1731
S S T N L P V L A L
e e e e b e b b b b
f     f s f   s

1741
L L L L Y G W S I T
b b b b b b b b b b
s s     s s s s   s

1751
P L M Y P A S F V F
b b b b b b b b b b
s   s s s s s     s

1761
K I P S T A Y V V L
e b e b e b b b b b
    f s f s s s   s

1771
T S V N L F I G I N
b b b b b b b b b b
  s   s s s s s s s

1781
G S V A T F V L E L
b b b b b b b b e b
  s     s s     f

1791
F T D N K L N N I N
b e e e e b e e b e
                  f
```

```
1801
DILKSVFLIF
ebbeebbbbb
   f

1811
PHFCLGRGLI
bbbbbbebbb
 s sssf ss

1821
DMVKNQAMAD
ebbeeebbbe
fs  ffs

1831
ALERFGENRF
bbeebeeeeb
     f

1841
VSPLSWDLVG
eeebebebbb
  f      s

1851
RNLFAMAVEG
ebbbbbbbeb
fs   s   s

1861
VVFFLITVLI
bbbbbbbbbb
      s

1871
QYRFFIRPRP
ebebbbeeee
f

1881
VNAKLSPLND
eeeeeeeeee

1891
EDEDVRRERQ
eeeebbeeee
ff fs  ff

1901
RILDGGGQND
ebeeeeeeee
f        f

1911
ILEIKELTKI
bbebeebeeb
ss    sff

1921
YRRKRKPAVD
eeeeeeeebe
f     ffsf

1931
RICVGIPPGE
ebbbbbeeee
  s  s   f

1941
CFGLLGVNGA
bbbbbbbeee
sssssssfff

1951
GKSSTFKMLT
eeeeeebebbe
fffffsfs f

1961
GDTTVTRGDA
eeeebeebeb
ff   f   s

1971
FLNKNSILSN
bbeeebbbee
     ss

1981
IHEVHQNMGY
beebeeebee
   sff sff

1991
CPQFDAITEL
beebebbeeb
sffsfs

2001
LTGREHVEFF
bebeebbebb
 fsffs

2011
ALLRGVPEKE
bebeeeeeee
s sffff

2021
VGKVGEWAIR
beebbebbbe
   s  s

2031
KLGLVKYGEK
ebbbbebbee
fs s

2041
YAGNYSGGNK
eeeeeeeeee
 ffffffff

2051
RKLSTAMALI
eebbebbbbb
ffssfs  ss

2061
GGPPVVFLDE
ebbbbbbbbe
f   s sssf

2071
PTTGMDPKAR
eeeeeeeebe
fffffff sf

2081
RFLWNCALSV
ebbbebbbbb
f s f

2091
VKEGRSVVLT
beebebbbbb
    fss ss

2101
SHSMEECEAL
bebbeebeeb
sf sffsffs

2111
CTRMAIMVNG
bebbbbbbeb
sfssssssfs

2121
RFRCLGSVQH
ebebeebbee
 s sffs ff

2131
LKNRFGDGYT
beeebeebbb
 f f ffss

2141
IVVRIAGSNP
bbbebeeeee
s  f

2151
DLKPVQDFFG
ebeebeebbe

2161
LAFPGSVLKE
eebeebebee
        ff

2171
KHRNMLQYQL
eeeebbeeeb
 f f sff

2181
PSSLSSLARI
eeebebbbeb
      s

2191
FSILSQSKKR
bebbbeeeee
s

2201
LHIEDYSVSQ
bebeeebebbe
    f fssf

2211
TTLDQVFVNF
eebeebbbeb
ffsffss fs

2221
AKDQSDDDHL
beeeeeeeeb
sf f

2231
KDLSLHKNQT
eebeeeeeeb

2241
VVDVAVLTSF
bbebbbbeeb

2251
LQDEKVKESY
beeeeeeeee
       ff

2261
V
e
f
```

The conservation scale:

?	1	2	3	4	5	6	7	8	9
Variable					Average				Conserved

e - An exposed residue according to the neural-network algorithm.

b - A buried residue according to the neural-network algorithm.

f - A predicted functional residue (highly conserved and exposed).

s - A predicted structural residue (highly conserved and buried).

X - Insufficient data - the calculation for this site was performed on less than 10% of the sequences.
