# Supplementary material for: Computational SNP Analysis and Molecular Simulation Revealed the Most Deleterious Missense Variants in the NBD1 Domain of Human ABCA1 Transporter
Source: Int J Mol Sci. 2020 Oct 14;21(20):7606. doi: 10.3390/ijms21207606 (PMC7589834; doi:10.3390/ijms21207606)
Supplement: Supplementary file 1 [file ijms-21-07606-s001.zip › Supplementary Files/Supplementary File 3.docx]

**Supplementary Material 3**


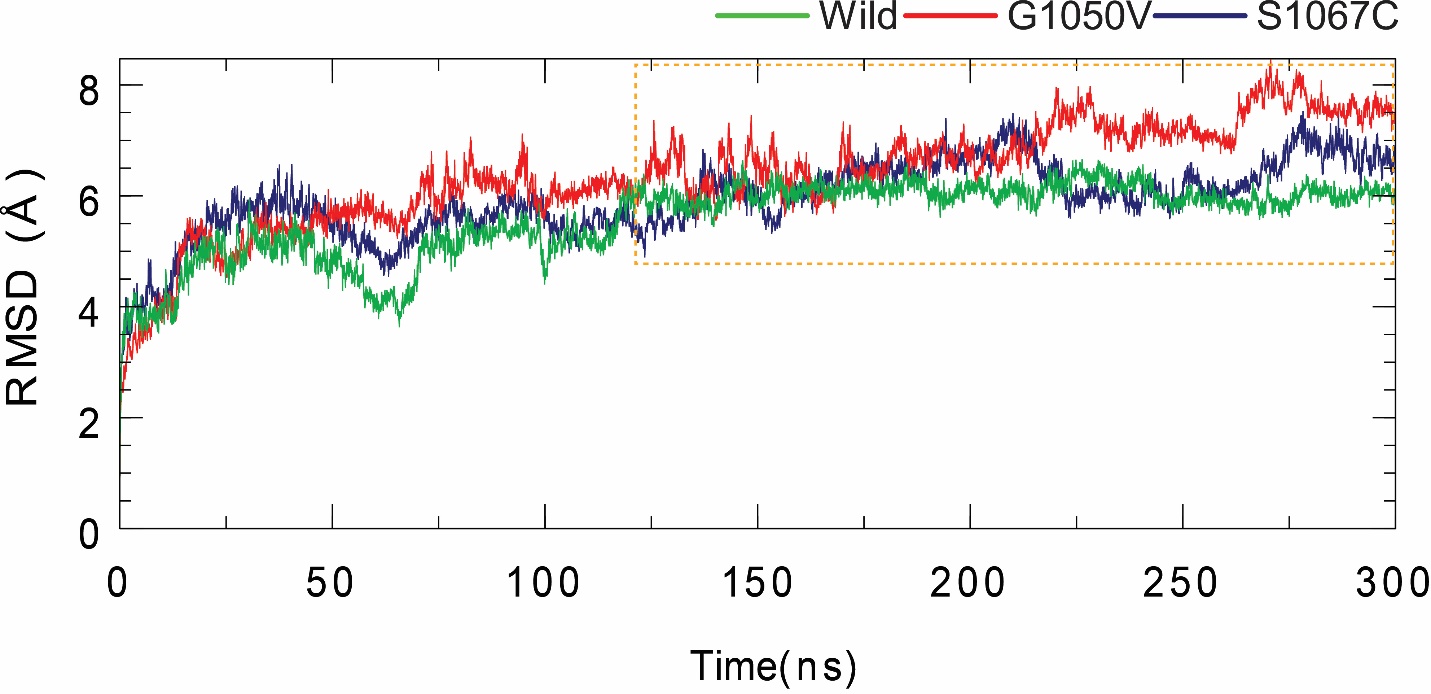


**Figure S1.** Analysis of Root-mean-square deviation (RMSD) based on the C*_α_* atoms for wild-type and NBD1 variants, which were calculated by comparing the starting structure of each simulation. Color in the plot, that is, green, red, dark blue, describes wild, G1050V, and S1067C, respectively. Trajectories marked by the dotted (orange color) box were considered for further analysis.


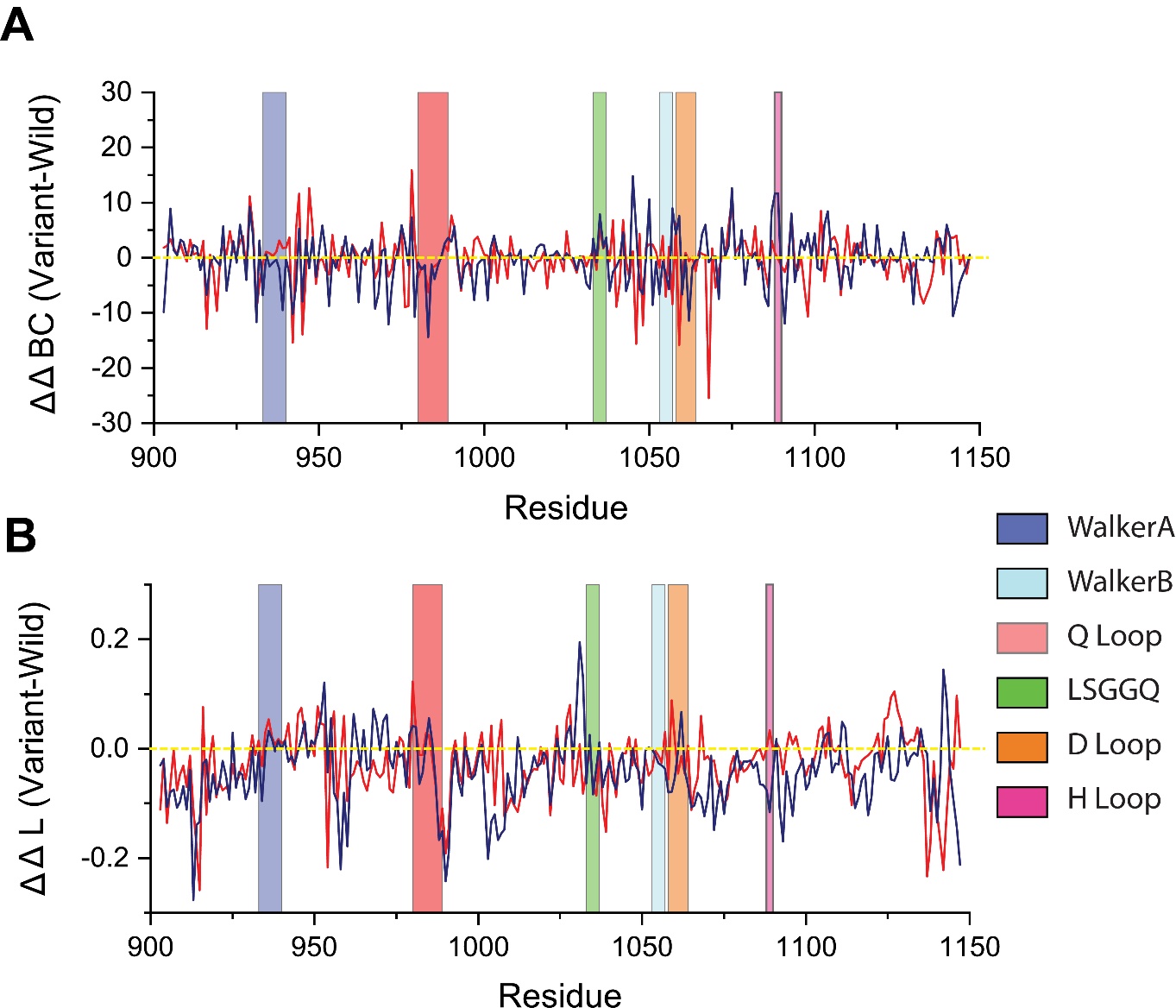


**Figure S2.** Effect of variants in the change of (A) average betweenness centrality and (B) average shortest path (variant minus wild). Line color in the plot, that is, red and dark blue describes G1050V and S1067C, respectively.
