# Supplementary material for: Computational SNP Analysis and Molecular Simulation Revealed the Most Deleterious Missense Variants in the NBD1 Domain of Human ABCA1 Transporter
Source: Int J Mol Sci. 2020 Oct 14;21(20):7606. doi: 10.3390/ijms21207606 (PMC7589834; doi:10.3390/ijms21207606)
Supplement: Supplementary file 1 [file ijms-21-07606-s001.zip › Supplementary Files/Supplementary File Caption.docx]

**Supplementary File Caption**

Supplementary File 1: Prediction of the effect of nsSNP by various tools.

Supplementary File 2: Conservation profile of amino acids in ABCA1 predicated by Consurf tool

Supplementary File 3: Results of molecular dynamics simulation, i.e., RMSD and dynamic residue network analysis.
